# Prefrontal cortex function and gait alterations during single- and dual-task walking in knee osteoarthritis

**DOI:** 10.1371/journal.pone.0331070

**Published:** 2025-09-11

**Authors:** Soyoung Lee, Ehyun Kim, Dilinazi Duolikun, Baekdong Cha, Claudio L. Ferre, Meryem Yücel, Deepak Kumar

**Affiliations:** 1 Department of Physical Therapy, Boston University, Boston, Massachusetts, United States of America; 2 Department of Occupational Therapy, Boston University, Boston, Massachusetts, United States of America; 3 Department of Biomedical Engineering, Boston University, Boston, Massachusetts, United States of America; Tehran University of Medical Sciences, IRAN, ISLAMIC REPUBLIC OF

## Abstract

Over-recruitment of the prefrontal cortex (PFC) during complex walking conditions may reflect altered motor and cognitive performance in people with knee osteoarthritis (OA). Our objectives were (1) to assess PFC activation, and motor and cognitive performance, during single- and dual-task walking in people with knee OA and (2) to examine the association of PFC activation with the performance. Forty-eight people with symptomatic knee OA completed three tasks, (1) single-task walking (STW) (2) subtraction by 7 from a 3-digit number (S7), and (3) dual-task walking (DTW), a combination of STW and S7. Oxygenated hemoglobin concentration changes (ΔHbO_2_) in bilateral prefrontal cortex (PFC) were assessed using functional Near-Infrared Spectroscopy. Motor performance outcomes included gait speed, step duration variability, and stride length variability. Cognitive performance was assessed as the correct response rate during S7. We used repeated measures ANCOVA to compare the outcomes by tasks. Correlation and multiple linear regression analyses were used to determine the association between PFC activation and performance outcomes. PFC activation was higher during STW and DTW compared to S7 but not significantly different between STW and DTW. People with knee OA walked slower (*d* = 0.63) and had higher variability in step duration (*d* = 0.45) and stride length (*d* = 0.37) during DTW compared to STW. Greater activation in right ventrolateral PFC (R^2^ = 0.15) and left dorsomedial PFC (R^2^ = 0.12) were associated with lower step duration variability. When walking is challenged with a cognitive task, people with knee OA show deterioration of gait performance and no change in PFC activation.

## Introduction

Knee osteoarthritis (OA) is a leading cause of pain and disability affecting approximately 600 Million people worldwide [1]. People with knee OA exhibit altered gait patterns (e.g., greater knee loading and excessive muscular co-contraction) likely reflecting alterations of underlying neuromotor control of gait [2,3]. Finding of greater gait variability in people with symptomatic knee OA provides evidence of altered neuromotor control [4,5]. Altered neuromotor control of gait and resulting gait deficits can increase the risk of falls, and worsening pain and physical function [6–10]. However, neural substrates underlying altered gait control in people with knee OA have not been examined, most likely due to the challenges in measuring brain activity during dynamic movement. This limitation has hindered the development of interventions that could directly target these neural mechanisms.

In contrast to the historical focus on spinal cord and midbrain as key regions for control of rhythmic locomotion, recent neuroimaging studies have highlighted the role of cortical activity for locomotor control [11,12]. Prefrontal cortex (PFC) is one of those regions, identified as crucial for goal-directed walking [12]. In other populations with gait impairments (e.g., Parkinson's Disease, stroke, multiple sclerosis), hyperactivation of the prefrontal cortex (PFC) during walking has been reported using functional near-infrared spectroscopy (fNIRS), a portable neuroimaging device, when compared to healthy controls [13–15]. Further, there is evidence of reduced “brain reserve,” i.e., the availability of neurophysiological resources to further increase PFC activity, when walking is challenged with concurrent motor or cognitive tasks (i.e., dual-task walking) [14,16]. A study by Bishnoi et al. demonstrated that individuals with knee OA showed a attenuated increase in PFC activation when transitioning from normal to perturbed walking, relative to healthy controls, suggesting limited neural resources during tasks requiring heightened neuromuscular control [17]. These findings are thought to reflect reliance on greater attentional resources during walking to compensate for pathological deficits (e.g., muscle weakness, proprioceptive loss) [3,14,18,19].

However, greater reliance on the PFC for performing tasks of moderate difficulty may reflect an inefficient neural mechanism, as proposed by the “neural efficiency theory”, which posits that higher brain activation is not inherently advantageous [20]. Possibly, the same level of task can be performed with lower cortical recruitment for young adults for example compared to older adults, which is more efficient neural processing [20]. However, for knee OA population, symptoms, such as pain, could induce neural inefficiency. A study by Hamacher et al. demonstrated that reducing pain severity in individuals with knee OA led to decreased motor-cognitive dual-task costs (toe-clearance variability) during dual-task walking suggesting that pain relief may enhance motor performance [21]. Therefore, understanding the neurophysiological mechanisms underlying gait control could provide new insight into existing interventions with the potential to restore normal movement patterns in knee OA populations.

Our primary objectives were (a) to characterize PFC activation and gait performance across single- and dual-task walking conditions in people with knee OA, and (b) to determine the association between PFC function and gait performance during single- and dual-task walking in people with knee OA. We hypothesized that (a) people with knee OA would exhibit worse gait performance but similar PFC activation during dual-task walking compared to single-task walking conditions due to reduced brain reserve, and (b) greater PFC activation during single- and dual-task walking would be associated with better gait performance in people with knee OA. A secondary aim was to examine differences in PFC function during single- and dual-task walking between people with knee OA and healthy controls.

## Methods

### 2.1. Participants

Participants were recruited from the general community using advertisements that included flyers posted in the community, and online and social media advertising from November 2022 to January 2024. Individuals were originally screed with online screening form and phone screening was followed up only for the ones who met the eligibility criteria. Inclusion criteria include (1) age between 50–75, (2) body mass index (BMI) < 40, (3) ability to walk for a minimum of 20 minutes without any assistive devices, (4) meeting National Institute for Health and Clinical Excellence clinical guidelines for knee osteoarthritis (i.e., age ≥ 50 years, presence of activity-related pain, presence of morning knee stiffness ≤ 30 minutes), (5) knee pain severity of ≥ 4/10 during the previous week, and (6) knee pain on most days for 3 months or more. Exclusion criteria include (1) mini-mental state examination (MMSE) score < 24, (2) contraindications to exercise, (3) any health conditions that limit the ability to walk (except knee pain), (4) currently receiving chemotherapy or radiation therapy for cancer except for non-melanoma skin cancer, (5) history of other disease that may involve the knee joint including inflammatory joint disease, (6) any knee surgery in the previous 6 months, (7) joint replacement in either hip or ankle, (8) previous knee osteotomy partial or total knee replacement in either knee, (9) corticosteroid or hyaluronic acid injections in either knee in the previous 3 months, (10) neurological conditions that impact motor functioning, and (11) other pain in lower back or legs that is greater than knee pain. Healthy controls (n = 10) were recruited after all OA participants had been enrolled and were frequency matched to the OA group based on age, sex, and BMI. Inclusion and exclusion criteria for the healthy individuals are provided in **Supplementary Table 1 in** S1 File.

The more painful knee, or a knee selected at random in case of similar pain in both knees, was designated as the index knee. For the control group, the study knee was selected at random. This study was approved by the Institutional Review Board at Boston University and all participants provided written informed consent before enrollment.

### 2.2. Experimental protocol

**Fig 1:**
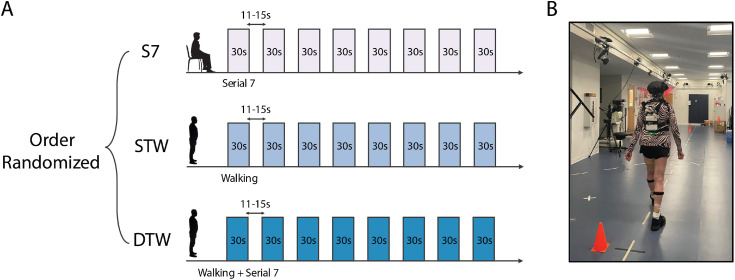
(A) Experimental protocol of Serial 7 subtraction (S7), single-task walking (STW), and dual-task walking (DTW) (B) Experimental setup during STW.

Participants performed three tasks during a single in-person visit (**Fig 1**). These tasks were: serial 7 subtraction (S7), i.e., serially subtract seven aloud from a random three-digit number while sitting, single-task walking (STW) i.e., walking around a 10m walkway at a self-selected pace in a counter-clockwise direction, and dual-task walking (DTW), i.e., walking around a 10m walkway while subtracting seven aloud from a random three-digit number at a self-selected pace in a counter-clockwise direction. Our dual task walking paradigm was designed to simulate daily walking, which often requires additional cognitive effort, such as walking while talking or navigating environmental challenges. The order of tasks was counterbalanced across participants. Each task consisted of eight 30 second bouts interspersed with a standing (for STW and DTW) or sitting (for S7) rest period that was randomly varied between 11-15s to avoid possible anticipation effects. The total duration of single run for each task was about 6 minutes. No guidance regarding task prioritization was provided for DTW.

### 2.3. fNIRS-derived PFC function

**Fig 2:**
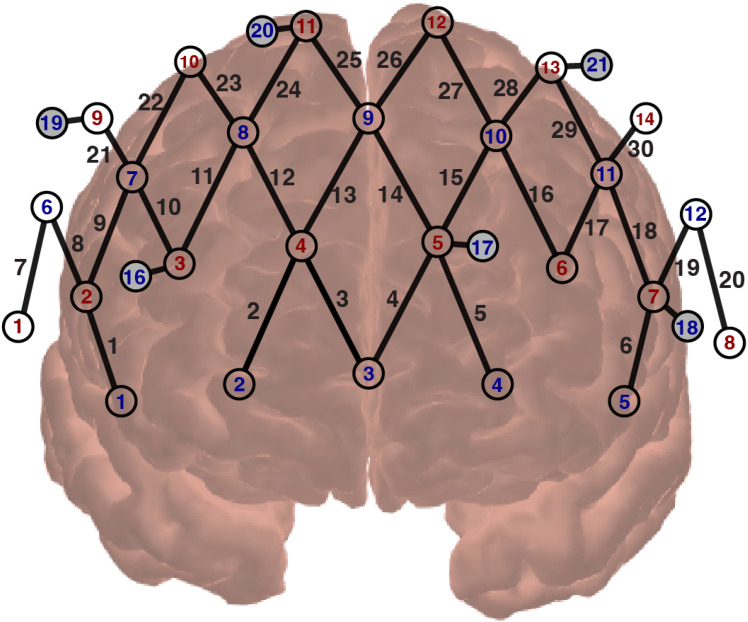
Probe design covering bilateral prefrontal cortex (PFC) areas. The probe consists of 14 sources (red) and 12 detectors (blue), resulting in 30 channels (numbered in black from 1 to 30); six short-separation detectors (colored in gray; numbered in blue from16 to 21) were used; PFC were subdivided into left ventrolateral PFC (LVLPFC) (Channel 6, 19, 20); right ventrolateral (RVLPFC) (Channel 1, 7, 8); left dorsolateral (LDLPFC) (Channel 16, 17, 18, 29, 30); right dorsolateral PFC (RDLPFC) (Channel 9, 10, 11, 21, 22); left dorsomedial (LDMPFC) (Channel 14, 15, 26, 27, 28); right dorsomedial (RDMPFC) (Channel 12, 13, 23, 24, 25); left ventromedial (LVMPFC) (Channel 4, 5); right ventromedial (RVMPFC) (Channel 2, 3).

We employed a continuous wave, wearable fNIRS system (NIRSport2; NIRx, Medical Technologies, Berlin, Germany) for data acquisition. Fourteen sources and twelve detectors, creating thirty long separation channels, were placed over the left-ventrolateral PFC (LVLPFC), right-ventrolateral PFC (RVLPFC), left-dorsolateral PFC (LDLPFC), right-dorsolateral PFC (RDLPFC), left-dorsomedial PFC (LDMPFC), right-dorsomedial PFC (RDMPFC), left-ventromedial PFC (LVMPFC), and right-ventromedial PFC (RVMPFC), as shown in **Fig 2**. Channels included in each subdivision of PFC are visualized in **Fig 2**. Sources and detectors are separated by approximately 30 mm (i.e., long-separation channels). Six short separation channels with an inter-optode distance of 8 mm were used to measure systemic hemodynamics over the superficial layer [22]. Near-infrared light was emitted at 760 and 850 nm, with a sampling rate of 10.2 Hz. Caps of varying sizes (54, 56, or 58 cm) were used based on head circumference, and a black shower cap was placed to minimize ambient light interference. Raw light intensities were collected through the software made by the manufacturer (Aurora fNIRS, NIRx, Medical Technologies)

The signal processing of fNIRS data was performed with Homer3 (v.1.58) [23]. Following the recent guideline [24,25], data was quality-checked and processed. Initially, channels exhibiting low intensity (<2e-03) or less than a certain threshold of signal to noise ratio (i.e., 5) were excluded. Subsequently, raw signals were converted to optical density (OD), and motion artifacts were corrected using spline interpolation and wavelet-based filtering methods [26,27]. The corrected OD signals underwent low-pass filtering using a sixth-order Butterworth filter with a 0.5 Hz cutoff frequency to eliminate slow drifts and high-frequency noise. Concentration changes of oxygenated hemoglobin (HbO₂) were calculated from the filtered OD signals using the modified Beer-Lambert law without applying partial pathlength correction [28]; hence, results are reported in μM·mm units. The hemodynamic response function (HRF) was estimated by a General Linear Model (GLM) approach that uses ordinary least squares. HRFs were modeled using consecutive Gaussian temporal basis functions with 1-second standard deviations, spaced at 1-second intervals, spanning from −2–30 seconds and accounting for third-order drift. To correct for systemic physiological interference, each long-separation channel was regressed against the short-separation channel with the highest temporal correlation. We had five participants having excessive motion artifacts in all the short-separation channels. For these individuals, we used the average value of the long-separation channels as a regressor. fNIRS data provide relative oxy-hemoglobin (HbO_2_) and deoxy-hemoglobin (HbR) concentration, indicators of the functional activity from the covered cortex regions. Given prior evidence suggesting greater sensitivity of HbO₂ compared to HbR for assessing locomotion-related cortical activity, we only reported HbO_2_ in the current study [29,30]. In the statistical analysis, we used the mean HRF of HbO_2_ from 5-30s period of each bout of each task after accounting for the 5s of physiological delay in the hemodynamic response [31].

### 2.4. Motor and cognitive performance

Gait performance outcomes included gait speed, variability in step duration, and variability in stride length during STW and DTW. To estimate gait speed and variability measures, participants wore three wireless inertial sensors (Opal, APDM, Portland, OR, USA; 128 Hz including tri-axial accelerometers, gyroscopes, and magnetic sensors), two at the dorsum of each foot, and one on the lower back. Real-time sensor data was recorded during walking and spatiotemporal gait measures were extracted for each bout of STW and DTW using manufacturer provided software (Moveo Explorer, APDM, Portland, OR, USA). The spatiotemporal measures (that included gait speed) were then used to calculate the variability measures. Specifically, coefficient of variation (CoV) of step duration and stride length were used as an indicator of gait variability. The CoV was calculated with the following equation: (Standard Deviation (SD)/Mean)*100. Cognitive performance was assessed with the correct response rate (%) (CRR = response rate per second × percent of accuracy) during serial 7 subtraction during S7 and DTW [32].

### 2.5. Statistical analysis

The normality of ΔHbO_2_ in each subregion of PFC and each performance outcome were determined visually with histogram comparison. Repeated measures Analysis of Covariance (ANCOVA), followed by pairwise comparisons, was used to compare ΔHbO_2_ in each subregion and each performance outcome between tasks with age, sex, and BMI as covariates. For each pairwise comparison, we report the mean difference (95% confidence intervals) and the effect size (Cohen’s *d*). Effect size of 0.2 can be considered a “small” effect size, 0.5 represents a “medium” effect size and 0.8 a “large” effect size [33]. We used Pearson correlations to examine the correlation between the ΔHbO_2_ of each subregion (8 regions) and each performance outcome. For pairs with a correlation coefficient (*r*) larger than 0.2, we further used multiple linear regression analysis with age, sex, and BMI as confounders. The normality of residuals from each model was tested using Q-Q (quantile-quantile) plot.

For the secondary analyses comparing OA and control groups, we used linear mixed-effects model to evaluate the interaction effect by groups (control vs. knee OA) and tasks (STW vs. DTW) on ΔHbO_2_ while adjusting for age, sex, and BMI as confounders. Partial eta-squared (ηp²) values were reported to quantify the magnitude of each fixed interaction effect. ηp² values were interpreted as small (0.01–0.05), medium (0.06–0.13), or large (≥0.14) effects [33]. If the interaction effects were not significant, we further evaluated the main effects for group (OA vs. control). We have reported the effect size (Cohen’s *d*) for each comparison. All statistical analyses were conducted using RStudio (version 2023.12.1 + 402).

## Results

Our sample comprised of 48 participants with knee OA and 10 control participants (Table 1). Our OA participants were over the age of 60, a majority were women, and their mean BMI was in the overweight category (Table 1). Based on the average Knee injury and Osteoarthritis Outcome Score (KOOS) scores, the participants were experiencing mild to moderate OA-related disability. The control group was on average, similar to the OA group in terms of age and proportion of women. However, the average BMI was lower for the control group.

**Table 1:** Participant characteristics.

	OA (n = 48)	Controls (n = 10)
Age, (years), Mean (SD)	64.8 (7.2)	62.6 (8.5)
Women, n (%)	35 (72.9)	7 (70.0)
BMI, kg/m^2^, Mean (SD)	29.5 (5.5)	25.9 (3.6)
Study knee at right side, n (%)	28 (58.3)	4 (40.0)
KOOS Pain (range 0–100), Mean (SD)	64.5 (12.1)	NA
KOOS ADL (range 0–100), Mean (SD)	73.1 (14.3)	NA
MMSE Score	28.8 (1.2)	28.9 (1.1)

BMI = body mass index; SD = standard deviation; KOOS = Knee injury and Osteoarthritis Outcome Score; ADL = Activity of daily living; MMSE = Mini-Mental State Examination.

### 3.1. Comparison of PFC activation and performance between tasks in the OA group

**Fig 3:**
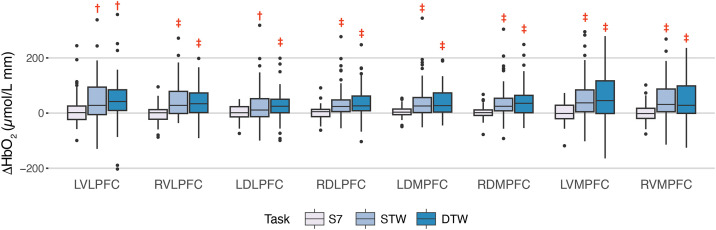
ΔHbO_2_ in each subregion of PFC during Serial 7 (S7), single-task walking (STW), and dual-task walking (DTW) in the OA group. †Medium effect size (*d*) when compared to S7; and ‡Large effect size (*d*) when compared to S7.

**Fig 4:**
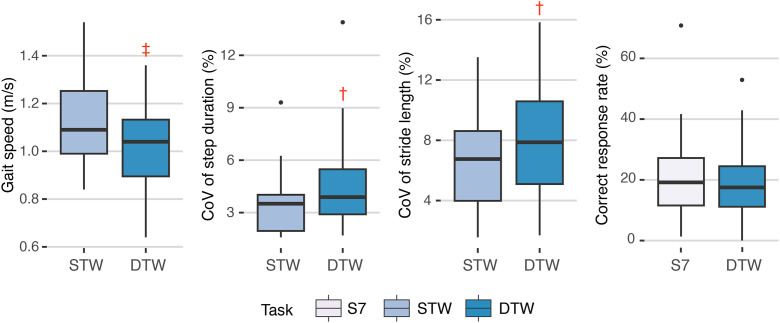
Boxplots of performance outcomes during Serial 7 (S7), single-task walking (STW) and dual-task walking (DTW) in the OA group; †Medium effect size (*d*); ‡Large effect size (*d*).

ΔHbO_2_ was similar between STW and DTW in all PFC subregions (Fig 3, Table 2). However, ΔHbO_2_ during STW and DTW was higher compared to S7 in all PFC subregions (Fig 3, Table 2). During DTW, OA participants had 9% (*d* = 0.63) slower gait speed and had 26% greater step duration CoV (*d* = 0.45) and 13% greater stride length CoV (*d* = 0.37) compared to STW (Fig 4, Table 3). The CRR was similar between S7 and DTW (*d* = 0.15).

**Table 2:** Post-hoc planned contrasts with an adjusted mean difference and effect size with 95% confidence intervals (CIs) for ΔHbO_2_ in all PFC subregions in the OA group.

Comparison	ΔHbO_2_	
	**Adjusted Mean difference (95% CI)**	**Cohen's *d*** **(95% CI)**
**LVLPFC**		
STW-S7	34.72 (9.81, 59.55)	0.46 (0.04, 0.87)
DTW-S7	29.07 (4.12, 54.01)	0.36 (−0.06, 0.77)
DTW-STW	−5.65 (−30.49, 19.26)	0.06 (−0.34, 0.47)
**RVLPFC**		
STW-S7	47.88 (30.47, 65.30)	0.87 (0.44, 1.30)
DTW-S7	46.66 (29.25, 64.07)	0.92 (0.49, 1.35)
DTW-STW	−1.22 (−18.64, 16.19)	0.02 (−0.39, 0.43)
**LDLPFC**		
STW-S7	26.01 (8.46, 43.56)	0.47 (0.06, 0.87)
DTW-S7	22.73 (5.18, 40.28)	0.51 (0.10, 0.91)
DTW-STW	−3.28 (−20.83, 14.27)	0.05 (−0.35, 0.45)
**RDLPFC**		
STW-S7	39.05 (23.21, 54.90)	0.81(0.39, 1.22)
DTW-S7	38.00 (22.15, 53.84)	0.79 (0.37, 1.20)
DTW-STW	−1.06 (−16.90, 14.79)	0.02 (−0.38, 0.42)
**LDMPFC**		
STW-S7	38.25 (21.98, 54.53)	0.72 (0.30, 1.13)
DTW-S7	31.82 (15.54, 48.10)	0.77 (0.35, 1.19)
DTW-STW	−6.43 (−22.71, 9.84)	0.10 (−0.30, 0.50)
**RDMPFC**		
STW-S7	42.24 (25.91, 58.57)	0.80 (0.38, 1.21)
DTW-S7	37.73 (21.40, 54.06)	0.79 (0.37, 1.21)
DTW-STW	−4.51 (−20.83, 11.82)	0.07 (−0.33, 0.47)
**LVMPFC**		
STW-S7	55.20 (28.95, 81.46)	0.82 (0.37, 1.26)
DTW-S7	53.18 (26.93, 79.44)	0.83 (0.38, 1.27)
DTW-STW	−2.02 (−28.27, 24.24)	0.02 (−0.40, 0.45)
**RVMPFC**		
STW-S7	50.35 (26.30, 74.41)	0.87 (0.42, 1.31)
DTW-S7	44.65 (20.60, 68.71)	0.71 (0.27, 1.14)
DTW-STW	−5.70 (−29.76, 18.35)	0.07 (−0.35, 0.50)

S7 and STW were used as reference conditions. left-ventrolateral PFC (LVLPFC); right-ventrolateral PFC (RVLPFC); left-dorsolateral PFC (LDLPFC); right-dorsolateral PFC (RDLPFC); left-dorsomedial PFC (LDMPFC); right-dorsomedial PFC (RDMPFC); left-ventromedial PFC (LVMPFC); and right-ventromedial PFC (RVMPFC); Single-task walking (STW); Dual-task walking (DTW); Serial 7 (S7). There were 2, 2, 1, 6, and 6 excluded participants from the comparison of LDLPFC, RDLPFC, LDMPFC, LVMPFC, and RVMPFC due to the missing values.

**Table 3:** Post-hoc planned contrasts with an adjusted mean difference for motor and cognitive performance outcomes between tasks in the OA group.

	Adjusted Mean difference (95% CI)	Cohen's *d* (95% CI)
Gait speed (m/s) _DTW-STW_	−0.11 (−0.14, −0.08)	0.63 (0.22, 1.04)
CoV of step duration (%) _DTW-STW_	0.88 (0.27, 1.50)	0.45 (0.05, 0.86)
CoV of stride length (%) _DTW-STW_	1.19 (0.27, 2.11)	0.37 (−0.04, 0.77)
S7 Correct response rate (%) _DTW-S7_	−1.76 (−3.48, −0.04)	0.15 (−0.25, 0.55)

S7 and STW were used as reference conditions. CoV = Coefficient of Variation.

### 3.2. Association of PFC activation with performance outcomes in the OA group

**Fig 5:**
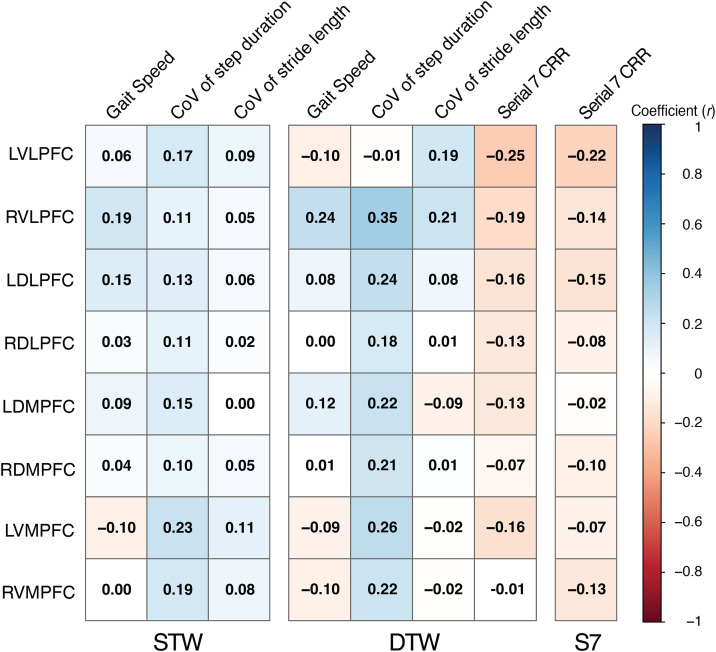
Heatmap of the Pearson correlation coefficient (*r*) between ΔHbO_2_ of each subregion and each performance outcome during single-task (STW/S7) and dual-task (DTW) in the OA group. Correlation coefficient (*r*) values were only overlayed in the figure. Coefficient of Variation (CoV) of step duration and stride length were negated so that positive values of all performance outcomes could indicate better gait performance. CRR = Correct response rate.

The heatmap in **Fig 5** visualizes the correlation coefficient between the ΔHbO_2_ of each region and each performance outcome by task. Multiple linear regression analyses were done with eleven correlation pairs of ΔHbO_2_ and performance outcomes with pairs having a correlation coefficient (*r*) greater than 0.2 (greater than a ‘very weak’ correlation) (Table 4). Greater ΔHbO_2_ in RVLPFC (R_2_ = 0.15) and LDMPFC (R_2_ = 0.12) were related with lower CoV (%) of step duration (i.e., lower variability) during DTW.

**Table 4:** Summary of multiple linear regression models determining the association between ΔHbO_2_ and motor/cognitive performance outcomes after adjusting for age, sex, and BMI in the OA group. Only pairs of ΔHbO_2_ and performance outcomes showing correlation coefficient (*r)* values greater than 0.2 were investigated. Bold indicates p < 0.05.

Exposure	Estimate per SD unit (95% CI)	Adjusted R^2^
***Associations with Gait Speed during DTW***		
RVLPFC	0.04 (−0.01, 0.09)	0.12
***Associations with CoV of Step Duration (%) during STW***		
LVMPFC	−0.31 (−0.85, 0.22)	0.04
***Associations with CoV of Step Duration (%) during DTW***	
**RVLPFC**	**−0.80 (−1.44, −0.16)**	**0.15**
LDLPFC	−0.57 (−1.21, 0.08)	0.10
**LDMPFC**	**−0.72 (−1.39, −0.05)**	**0.12**
RDMPFC	−0.64 (−1.29, 0.02)	0.11
LVMPFC	−0.64 (−1.33, 0.05)	0.15
RVMPFC	−0.59 (−1.28, 0.09)	0.11
***Associations with CoV of Stride Length (%) during DTW***	
RVLPFC	−0.78 (−1.75, 0.18)	0.19
***Associations with CRR during *** *S7*		
LVLPFC	−2.70 (−6.47, 1.08)	0.00
***Associations with CRR during DTW***	
LVLPFC	−2.66 (−5.82, 0.50)	0.13

left-ventrolateral PFC (LVLPFC); right-ventrolateral PFC (RVLPFC); left-dorsolateral PFC (LDLPFC); right-dorsolateral PFC (RDLPFC); left-dorsomedial PFC (LDMPFC); right-dorsomedial PFC (RDMPFC); left-ventromedial PFC (LVMPFC); and right-ventromedial PFC (RVMPFC), Coefficient of Variation (CoV), Single-task walking (STW), Dual-task walking (DTW), Serial 7 (S7).

### 3.3. Secondary analysis: Comparison of PFC activation between OA and control groups

There was a significant interaction between group and task for LDMPFC ΔHbO_2_ with small effect size (β = 38.70, p = 0.040, ηp² = 0.04). Individuals with knee OA exhibited greater ΔHbO_2_ during STW vs. S7 but the ΔHbO_2_ was similar across the two tasks for controls. (**Supplementary Fig 1 in** S1 File). No significant interaction effects were observed for ΔHbO_2_ in other subregions of PFC. For these other subregions, people with knee OA showed higher ΔHbO_2_ with small to medium effect sizes (*d* = 0.2–0.4) (**Supplementary Fig 1 in** S1 File).

## Discussion

We investigated the role of the prefrontal cortex during STW and DTW in people with knee OA. We identified three key findings in our cohort of people with symptomatic knee OA: (1) PFC activation was similar between STW and DTW (2) walking performance was worse during DTW compared to STW, but cognitive performance was similar between DTW and the S7; and (3) greater PFC activation during DTW was associated with lower gait variability. Additionally, we observed that people with knee OA had generally greater PFC activation compared to healthy controls. These novel findings indicate that people with knee OA may lack the ability to harness PFC to maintain gait performance during challenging walking conditions.

### 4.1. Comparison of PFC activation between tasks in the OA group

We observed similar activation in all PFC subregions during STW and DTW in our cohort of people with knee OA. Given the greater cognitive demand during DTW compared to STW, an increase in PFC activation would be expected as reported in prior studies among healthy individuals [16,34,35]. Bishnoi et al. reported an increase in PFC activation during a perturbed walking task (i.e., anterior-posterior perturbation on a treadmill) compared to normal walking, in people with knee OA, which differs from our findings [17]. This discrepancy may be primarily attributed to differences in task characteristics and disease severity. For individuals with knee OA, perturbed walking may be a more attention-demanding task than mental arithmetic. The absence of increased PFC activation in our study may indicate reduced brain reserve [36]. Evidence for this comes from prior studies in other populations with gait impairments. Hawkins et al. reported a significant increase in PFC activation from STW to DTW in healthy young and older adults but not in people with stroke [14]. Similar findings were reported in people with Parkinson's disease [13,37]. These observations are consistent with the Compensation-Related Utilization of Neural Circuits Hypothesis (CRUNCH) hypothesis [18]. According to CRUNCH, older adults exhibit increased brain activation relative to young adults when faced with submaximal levels of task demand, which serves to preserve task performance. However, this increased activation brings them closer to their brain’s resources limit, which has been interpreted as a ‘reduced brain reserve mechanism’. As task demands increase, neurophysiological resources reach a limit, resulting in insufficient processing and diminished task performance.

Our findings from comparison of PFC activation between people with knee OA and age- and sex-matched healthy older adults support the notion of suboptimal neural efficiency in the OA group. We observed higher PFC activation in the OA group irrespective of task suggesting relatively greater remaining cognitive resources in healthy individuals (**Supplementary Fig 1 in** S1 File). Additionally, although not statistically analyzed, the control participants showed increased activation in the left hemisphere and a concurrent decrease in the right hemisphere from STW to DTW, a pattern not seen in the OA group (**Supplementary Fig 1 in** S1 File). This pattern may reflect a more efficient reallocation of neural resources toward the left hemisphere, consistent with the neural efficiency hypothesis, which posits that more cognitively efficient individuals exhibit less diffuse and more targeted brain activation during cognitive tasks [20]. This finding aligns with previous literature demonstrating left-lateralized activation during mental arithmetic and dual-task paradigms [38,39].

We observed significantly lower activation across all PFC subregions during the S7 task compared to both STW and DTW. This finding is consistent with recent evidence in individuals with Parkinson's disease [37]. However, in the data from healthy individuals, similar activation levels were observed in some PFC subregions between S7 and walking tasks. Taken together, these results suggest that motor performance may be more attention-demanding than cognitive tasks alone in individuals with chronic pain or motor impairments, such as those with knee OA, compared to healthy older adults. Clinically, this underscores the importance of promoting gait automaticity to reduce executive load and lower fall risk in the knee OA population.

### 4.2. Comparison of performance outcomes between tasks

In our study, people with knee OA walked slower and had increased variability in step duration and stride length during DTW compared to STW. We can deduce that our dual-task walking paradigm had induced adequate cognitive loading during walking given that a 0.1m/s reduction in gait speed is a clinically meaningful change [40]. However, the cognitive performance, assessed with the correct response rate during serial 7 subtraction, was similar during DTW and S7. These findings collectively indicate that people with knee OA might have prioritized the cognitive task during DTW given the deterioration in gait performance compared to STW, but similar cognitive performance compared to S7. Similar findings have been observed from people with neurological disease (i.e., Parkinson's Disease, stroke) [14,37], chronic back pain and older adults with dementia, who showed decreased gait speed and increased gait variability during dual-task overground walking compared to single-task overground walking [41,42]. Meanwhile, prior studies consistently show similar performance for the cognitive task during single- and dual-task conditions [37,43–45]. These findings underscore the importance of gait training in dual-task condition for people with knee OA since the dual-task cost (i.e., increased gait variability) could potentially be reduced by such training [46]. Hence, interventions to improve dual-task walking performance may be considered for reducing fall risk and improving gait quality in people with knee OA.

### 4.3. Association between PFC activation and performance outcomes

In people with knee OA, we observed that greater RVLPFC and LDMPFC activation was related with lower gait variability, but only during DTW. Similar relationships were seen for other PFC subregions but it is important to note that, as would be expected, all associations between PFC activation and gait measures were weak with adjusted model R^2^ ≤ 0.15. However, the consistency of associations across the PFC subregions suggests that individuals with knee OA may need greater executive resources to prevent a deterioration in gait performance. Similar results were seen for people with Parkinson's Disease where greater DLPFC activity was related to lower variability in stride length and cadenced during walking while counting forward [37]. A significant positive correlation between better functional mobility (based on Berg Balance Testing) and higher PFC activation in people with hemiplegic stroke also supports our results [47]. Interestingly, in healthy individuals higher DLPFC activity has been reported to be associated with higher gait variability and worse cognitive performance [34]. These contrasting findings may reflect the inefficiency of PFC function in individuals with knee OA. Unlike healthy individuals who can perform walking tasks with minimal cortical demand, those with knee OA appear to rely more heavily on top-down control from the PFC, as suggested by comparisons between the two groups. This pattern is consistent with the concept of neural inefficiency, in which increased brain activation does not necessarily lead to improved performance but may instead indicate reduced gait automaticity and greater cognitive burden [20]. Clinically, such inefficiency may limit adaptability to complex walking environments and contribute to an increased risk of instability or falls during multitasking. These findings emphasize the value of incorporating dual-task assessments into clinical evaluations and support the potential benefits of interventions aimed at enhancing neural efficiency through targeted motor-cognitive training in this population.

We should note the limitations of our study that may impact interpretation. Our study was cross-sectional, and therefore, we cannot confirm causality or directionality. Our findings may not be generalizable to individuals with knee OA who may have different clinical characteristics compared to our cohort. We also need to acknowledge that we cannot rule out the risk of type II error although adjustments for multiple comparisons are not recommended if associations are biologically plausible [48]. Our interpretations are based on observed effect sizes which are independent of the study small size unlike p-values. Though much research using fNIRS during walking examines the prefrontal cortex, it is important to note that other cortical areas are likely involved in executive locomotor control. Previous studies have shown changes in motor and somatosensory regions during walking [29,49,50]. Future research should explore a broader range of cortical regions. Finally, we did not include a control group in our study. However, inclusion of a control group is unlikely to provide any clinically meaningful information given that our focus is on a population with knee OA and there are several prior studies in healthy cohorts.

## Conclusion

In conclusion, the current study highlights that individuals with knee osteoarthritis have similar prefrontal cortex activation during single- and dual-task walking. Further, their gait performance is worse during dual-task walking compared to single-task walking but cognitive performance is similar during dual-task walking and serial 7 task. Lastly, greater prefrontal cortex activation during dual-task walking was related to lower gait variability. Overall, these findings suggest that people with knee OA may not have sufficient brain reserve to increase prefrontal cortex function and maintain gait performance when challenged with dual-task conditions during walking. This may increase their risk of falls while walking in everyday life and interventions to improve dual-task walking performance may be considered in future studies.

## Supporting information

S1 File (DOCX)
