# Supplementary material for: Prefrontal cortex function and gait alterations during single- and dual-task walking in knee osteoarthritis
Source: PLoS One. 2025 Sep 11;20(9):e0331070. doi: 10.1371/journal.pone.0331070 (PMC12425207; doi:10.1371/journal.pone.0331070)
Supplement: S1 File — (DOCX) [file pone.0331070.s001.docx]

**Supplementary Table1. Inclusion and exclusion criteria for healthy control individuals.**

| **Inclusion Criteria** | **Exclusion Criteria** |
| --- | --- |
| - Age between 50-75years - BMI<40 - Ability to walk for a minimum of 20 minutes without any assistive device - Can speak and understand English at a sufficient level to understand the study procedures and informed consent. | - Mini-mental state examination (MMSE) score < 24 - Contraindications to exercise - Any health condition that limits ability to walk (except knee pain for OA participants) - Currently receiving chemotherapy or radiation therapy for cancer except non-melanoma skin cancer - History of other disease that may involve the index joint including inflammatory joint disease such as rheumatoid arthritis, seronegative spondyloarthropathy (eg, ankylosing spondylitis, psoriatic arthritis, inflammatory bowel disease related arthropathy), crystalline disease (eg, gout or pseudogout), lupus erythematosus, knee joint infections, Paget’s disease affecting the knee, or knee joint tumors. - Any knee surgery in the previous 6 months - Joint replacement in either hip or ankle - Previous knee osteotomy partial or total knee replacement in either knee - Corticosteroid or hyaluronic acid injections in either knee in the previous 3 months - Neurological conditions that impacts motor functioning (e.g., stroke, Parkinson’s disease, Alzheimer’s disease, Multiple Sclerosis, diabetic neuropathy, etc). - Pregnancy (self-report) - Participation in another clinical trial for any joint or muscle pain - Suspected or known drugs or alcohol abuse - Wrist fracture in both wrists within past 6 months (exclusion applies only to PPT testing) - Myocardial infarction within the past 6 months (exclusions applies only to CPM testing) - History of Raynaud’s syndrome, active vasculitis, or peripheral vascular disease. (exclusions applies only to CPM testing) - history of lymphedema, Takayasu’s arteritis, or arteriovenous fistula for hemodialysis in both arms (exclusion applies only to CPM testing) - History of knee pain in past 12-months - History of diagnosis of knee osteoarthritis - Any current musculoskeletal pain - Any lower-limb or lower back injuries in the previous 3-months |

**Supplementary Figure 1**. **HbO_2_ in each PFC subregion between the knee OA and control groups during S7, STW and DTW. Dot and error bar denote mean and one standard error, respectively. ‡Medium effect size (*d*) in between-group difference.**
